# Comparison of aortic stiffness and hypertension in repaired coarctation patients with a bicuspid versus a tricuspid aortic valve

**DOI:** 10.1186/s12968-023-00941-0

**Published:** 2023-06-15

**Authors:** Kwannapas Saengsin, Kimberlee Gauvreau, Ashwin Prakash

**Affiliations:** 1 Department of Cardiology, Boston Children’s Hospital, 300 Longwood Avenue, Boston, MA 02115 USA; 2 Department of Pediatrics, Harvard Medical School, Boston, MA USA

**Keywords:** Coarctation of the aorta, Bicuspid aortic valve, Vascular stiffness, Cardiac MRI, Aortic dilation

## Abstract

**Background:**

Coarctation of the aorta (COA) is associated with reduced aortic distensibility and systemic hypertension (HTN). 60–85% of COA patients have a bicuspid aortic valve (BAV). It is not known if the presence of a BAV accentuates the aortopathy and HTN in CoA patients. We examined whether patients with COA and a BAV had lower aortic distensibility by CMR, and a higher prevalence of systemic HTN compared with COA patients with a tricuspid aortic valve (TAV).

**Methods:**

In successfully repaired COA patients excluding those with residual COA, ascending aorta (AAO) and descending aorta (DAO) distensibility was calculated by CMR. HTN was assessed using standard pediatric and adult criteria.

**Results:**

Among 215 COA patients (median age 25.3 years), 67% had a BAV, and 33% had a TAV. Median AAO distensibility z-score was lower in the BAV group (− 1.2 versus − 0.7; p = 0.014) but DAO distensibility was similar in BAV and TAV patients. HTN prevalence was similar in BAV (32%) and TAV groups (36%, p = 0.56). On multivariable analysis controlling for confounders, HTN was not associated with BAV but was associated with male gender (p = 0.003) and older age at follow-up (p = 0.004).

**Conclusions:**

In young adults with treated COA, those with a BAV had stiffer AAO compared to those with a TAV, but DAO stiffness was similar. HTN was not related to BAV. These results suggest that although the presence of a BAV in COA exacerbates the AAO aortopathy, it does not exacerbate the more generalized vascular dysfunction and associated HTN.

## Introduction

Coarctation of the aorta (COA) is a common form of congenital heart disease with an incidence of 1 in 3000–4000 live births [1, 2]. COA is not a simple mechanical narrowing and is associated with reduced aortic distensibility and generalized vascular dysfunction, which leads to a high prevalence of systemic hypertension, left ventricular hypertrophy, ischemic heart disease, and stroke despite successful repair [3–12]. Reduced aortic distensibility has been associated with higher aorto-carotid wave transmission, which may contribute to cerebral aneurysms and stroke [9]. 60–85% of patients with COA have a bicuspid aortic valve (BAV) [13–16]. BAV patients also have reduced ascending aortic (AAO) distensibility, independent of COA [17, 18]. Since COA and BAV are both associated with abnormal vascular properties, it is not known if the presence of a BAV accentuates the reduced AAO distensibility seen in COA and if this additional abnormality increases the already high risk of systemic hypertension, compared to COA patients with a BAV. Therefore, we examined whether patients with COA and a BAV had lower aortic distensibility by CMR, and a higher prevalence of systemic hypertension compared with COA patient with a tricuspid aortic valve (TAV).

## Methods

A retrospective review of existing clinical data at Boston Children’s Hospital from January 2005 until December 2019 was performed. Demographic, clinical, and surgical data were abstracted from the medical records. The Department of Cardiology’s Scientific Review Committee and the Boston Children’s Hospital’s Committee on Clinical Investigation approved this retrospective review of existing clinical data and waived the requirement for informed consent.

### Subjects

Children and adults with COA who had undergone CMR between 2005 and 2019 with available images to measure aortic distensibility were included. Subjects with the following were excluded: unicuspid aortic valve, associated complex congenital heart defects (aside from simple septal defects and patent ductus arteriosus), genetic syndromes, connective tissue disorder, significant recurrent COA (upper to lower extremity systolic blood pressure gradient > 20 mm Hg), severe aortic valve stenosis (by echocardiography, within 1 year of the CMR), severe aortic regurgitation (CMR regurgitation fraction > 40%), and history of surgery involving the aortic root or ascending aorta. Sievers classification was used to describe the types of BAV [19]. For patients who had reintervention on the aortic arch, classification of the type and age at repair was based on the first arch intervention.

### CMR imaging

CMR examinations were performed for clinical indication using a commercially available whole-body scanner (Achieva; Philips Healthcare, Best, The Netherlands). When subjects had multiple available examinations, the most recent examination was used for analysis. In young patients who could not cooperate with breathing instructions, the examination was performed under general anesthesia. Brachial artery blood pressure was measured in the right arm before each examination in the supine position using commercial oscillometric blood pressure recorders. Electrocardiogram (ECG)-gated 2-dimensional cine steady-state free precession imaging of the left ventricular outflow tract in 2 orthogonal planes was performed that were then used to plan a stack of cine steady-state free precession (SSFP) images in the short axis of the AAO and the descending aorta (DAO) as previously described. Aortic valve morphology (BAV or TAV) was determined on a stack of cine SSFP images in the short axis of the aortic root.

### CMR image analysis

**Fig. 1:**
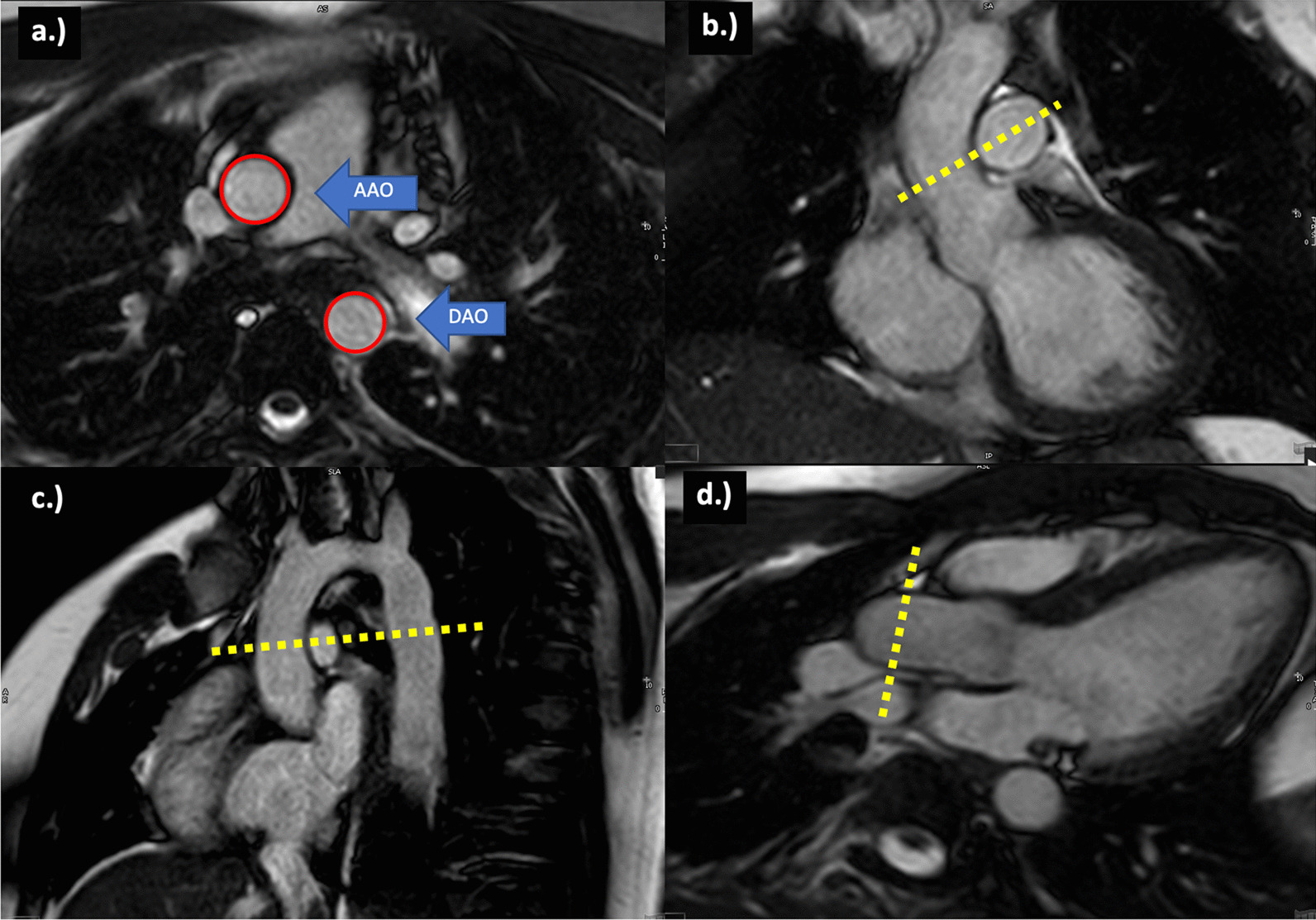
**a** Planimetry of the AAO and DAO to measure the CSA in both systole and diastole; **b** Cross-reference synchronized image of the oblique coronal left ventricular outflow tract used to select the slice that crosses the AAO at the widest perpendicular point at the level of the right pulmonary artery; **c** Cross-reference synchronized image of the oblique sagittal long-axis aortic arch perpendicular point at the level of the right pulmonary artery; **d** Cross-reference synchronized image of left ventricular outflow tract long-axis. *AAO* ascending aorta, *CSA* cross-sectional area, *DAO* descending

Cine steady-state free precession CMR images were analyzed at 2 locations (AAO and thoracic DAO, Fig. 1) to calculate parameters of stiffness as previously reported [17, 20]. At each location, the cross-sectional area was measured by a single observer using manual planimetry at both peak systole and end-diastole. Images were cross-referenced with 2 long axis planes to select the appropriate short-axis slice perpendicular to the aorta. Aortic stiffness was assessed using the following parameters as previously described [20, 21].

$$Strain=\frac{\mathrm{Systolic \, area }-\mathrm{ Diastolic \, area}}{\mathrm{Diastolic \, area}}$$

$$Distensibility=\frac{\mathrm{Strain}}{\mathrm{Brachial \, pulse \, pressure}}$$

$$\beta \, stiffness \, index=\frac{\mathrm{ln}(systolic \, blood \, pressure/diastolic \, blood \, pressure)}{\mathrm{Strain}}$$

Images were analyzed using commercially available software Cvi^42^ version 5.10 (Circle Cardiovascular Imaging Inc. Calgary, AB, Canada). For AAO distensibility data, *z* scores were calculated using previously published normative data reported by Voges et al. [22].

### Blood pressure measurements

All available blood pressure recordings following repair were abstracted from the patient’s medical record. Following standard practice at our institution, right arm blood pressure (BP) was recorded while seated using commercial oscillometric blood pressure devices and size-appropriate cuffs. Four-extremity BP was recorded in the supine position. Arm-leg BP difference was calculated as the difference between the systolic (SBP) in the right arm and the leg with the higher SBP. The most recent recording of arm-leg BP difference was used to exclude patients with significant residual coarctation. Patients satisfying standard pediatric (for patients < 18 years of age: systolic and/or diastolic BP ≥ 95th percentile for age, gender and height) or adult (for patients ≥ 18 years of age: systolic BP ≥ 130 and/or diastolic BP ≥ 80 mm Hg) criteria for resting right arm hypertension on 2 separate outpatient visits were labeled as having systemic hypertension [23, 24]. Patients currently on anti-hypertensive medication were classified as having hypertension only if they satisfied standard criteria for hypertension on 2 separate outpatient visits prior to initiation of treatment. Patients who had transient post-procedure hypertension (with or without antihypertensive treatment), that resolved with normal BP recordings on at least two subsequent outpatient visits (without antihypertensive treatment) were not classified as having hypertension. For the purposes of analysis, patients with well-controlled hypertension were treated the same as those with poorly controlled or newly diagnosed hypertension.

### Statistical analysis

Wilcoxon rank-sum test was used to compare aortic stiffness parameters between COA patients with BAV and COA patients with TAV. Logistic regression models using hypertension as the outcome and predictor variable BAV were fit with and without the adjustment for AAO and DAO distensibility to examine whether the distensibility measures affected these relationships. Statistical analysis was performed using commercially available software (Stata version 15.0; StataCorpLP, College Station, Texas).

## Results

### Subjects

**Fig. 2:**
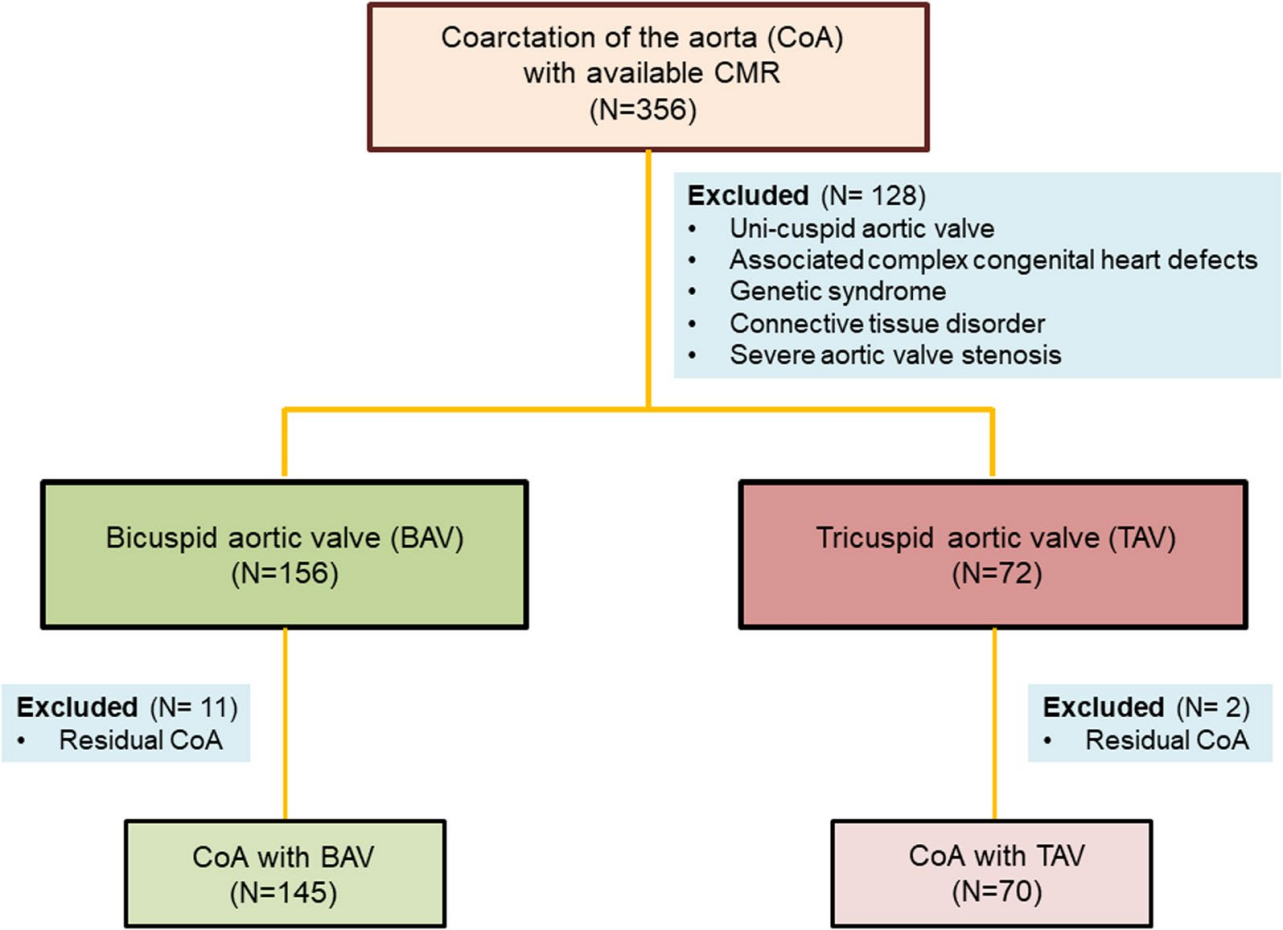
Summary of included and excluded patients

Details of included and excluded subjects are summarized in Fig. 2. Subject characteristics of included patients are summarized in Table 1. The study population consisted of children and young adults, with a median age of 25.3 years at last follow-up (interquartile range (IQR): 18.8, 34.3). As expected, a majority (67%) of patients had a BAV. Subjects with BAV and TAV were similar with respect to most characteristics including gender, age and type of repair, age at follow-up, and medication use. As expected, aortic stenosis and regurgitation were more common in the BAV group and BAV patients had a larger median AAO diameter *z-*score. 56/215 (26%) subjects had at least one reintervention on their aortic arch (transcatheter or surgical). The majority of subjects in both groups were on antihypertensive medication, with only minor differences in medication use between groups.

**Table 1:** Patient characteristics

	All Patients (n = 215)	COA with BAV (n = 145)	COA with TAV (n = 70)	p value
Age, years	25.3 (18.8, 34.3)	25.7 (1.3,34.3)	24.5 (17.7, 34.3)	0.75
Gender, male, n (%)	121 (56)	85 (59)	36 (51)	0.38
BSA, m^2^	1.8 (1.6, 2)	1.8 (1.6, 2)	1.8 (1.6, 2)	0.89
Type of BAV, n (%)				
Left-right fusion		125 (86)		
Right-non fusion		17 (12)		
Left-non fusion		3 (2)		
Age at COA treatment, years	1.7(0.1, 6.9)	1.1(0.1, 1.1)	2.4 (0.1, 10.2)	0.37
Treatment modality, n (%)				0.72
Surgery	183 (85)	125 (86)	58 (84)	
Balloon dilation	18 (8)	12 (8)	6 (9)	
Stent	14 (7)	8 (6)	6 (9)	
Type of surgical repair, n (%)				0.37
End-to-end repair	107 (59)	77 (62)	30 (51)	
Subclavian flap	25 (14)	15 (12)	10 (17)	
Patch reconstruction	13 (7)	6 (5)	7 (12)	
Interposition graft	8 (4)	5 (4)	3 (5)	
Extended end-to-end repair	1 (0.5)	1 (1)	0	
Unavailable surgical record*	29 (16)	21 (17)	8 (14)	
> Mild aortic regurgitation, n (%)	11(5)	11 (8)	0	0.02
> Mild aortic stenosis, n (%)	7 (3)	7 (5)	0	0.10
Medication use, n (%)				
β-blocker	33 (16)	18 (12)	15 (21)	0.11
ACE inhibitor	43 (20)	28 (19)	15 (21)	0.72
Hydrochlorothiazide	10 (5)	9 (6)	1 (1)	0.17
Angiotensin receptor blocker	9 (4)	9 (6)	0	0.03
Calcium channel blocker	8 (4)	5 (3)	3 (4)	0.72

Data are presented as number (percent) or median (IQR range)

*BSA* body surface area, *BAV* bicuspid aortic valve, *COA* Coarctation of the aorta, *ACE* angiotensin-converting enzyme, *TAV* tricuspid aortic valve

*In a minority of patients whose initial surgical repair was at another institution, surgical records were not available to confirm details

### Aortic stiffness parameters

**Fig. 3:**
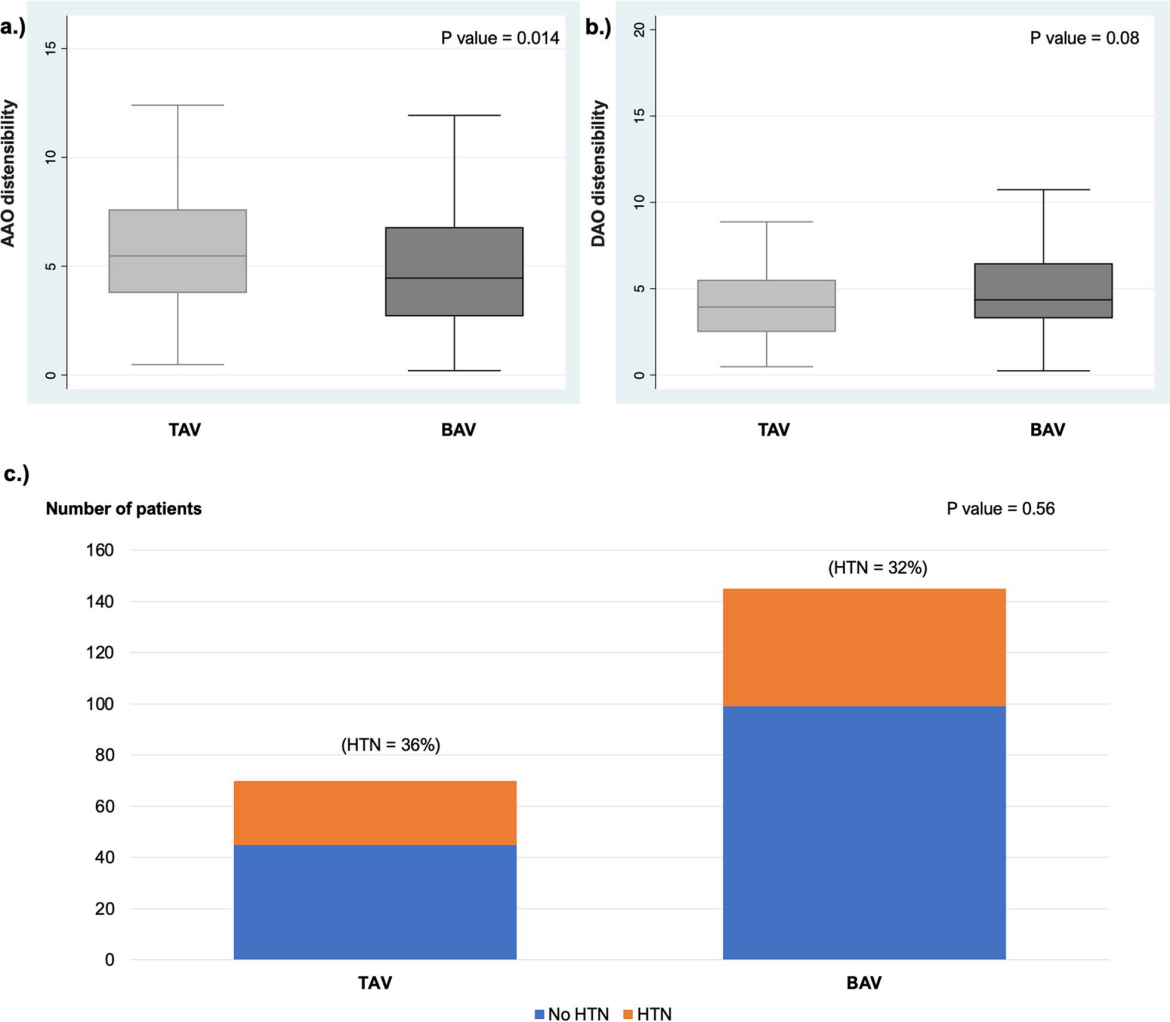
**a** Box plot comparing AAO distensibility in BAV and TAV patients; **b** Box plot comparing DAO distensibility in BAV and TAV patients; **c** Bar plot comparing prevalence of HTN in BAV and TAV patients. *AAO* ascending aorta, *BAV* bicuspid aortic valve, *DAO* descending, *TAV* tricuspid aortic valve

CMR derived aortic stiffness parameters at the AAO and DAO are summarized in Table 2 and Fig. 3. BAV patients had a stiffer AAO compared to TAV patients. However, at the DAO, the differences in stiffness between groups were less prominent with similar distensibility values.

**Table 2:** Comparison of hypertension and aortic stiffness parameters between groups

	COA with BAV (n = 145)	COA with TAV (n = 70)	p value
Hypertension	46 (32%)	25 (36%)	0.56
AAO stiffness parameters			
Strain (%)	26.3 (15.7, 37.1)	33.3 (21.6, 44.7)	**0.011**
Distensibility (× 10^–3^ mmHg^−1^)	4.5 (2.7,6.8)	5.5 (3.8, 7.6)	**0.014**
Distensibility z-score	− 1.2(− 3.0,− 0.3)	− 0.7(− 2.1, 0.4)	**0.001**
β Stiffness Index	2.5 (1.7,4)	2.0 (1.5, 2.8)	**0.010**
DAO stiffness parameters			
Strain (%)	26.9 (19.2, 35.8)	22.3(15.8, 29.7)	**0.034**
Distensibility (× 10^–3^ mmHg^−1^)	4.4(3.3, 6.5)	3.9(2.5, 5.5)	0.08
β Stiffness index	2.4 (1.8, 3.4)	2.8 (2.0, 4.2)	0.05

Aortic stiffness parameters have been measured at 2 locations (AAO and DAO). Significant p values are in bold for Wilcoxon ranksum test. Values are expressed as median (interquartile range) or number (%)

*AAO* Ascending aorta, *DAO* descending aorta, *CMR* Cardiac magnetic resonance, *CSA* cross-sectional area

### Hypertension

The overall prevalence of hypertension was 33% and as seen in Table 2 and Fig. 3, the prevalence of hypertension was similar in BAV and TAV patients. As seen in Table 3, factors associated with hypertension on univariate analysis included male gender, age at follow-up, age at treatment, lower DAO strain, and lower DAO distensibility, but not BAV. On multivariable analysis (Table 4), we explored two separate models, one including AAO distensibility *z-*score, and the other including DAO distensibility. In both models, no association was found between BAV and hypertension, even after controlling for possible confounders. The only factors independently associated with hypertension included male sex and older age at follow-up.

**Table 3:** Univariate analysis of factors associated with systemic hypertension

	Odds ratio	95% CI	p Value
Age at MRI	1.03	(1.02–1.06)	**0.001**
Age at CoA treatment	1.003	(1.001–1.006)	**0.011**
Sex (male)	2.22	(1.22–4.06)	**0.009**
BAV	0.83	(0.46–1.52)	0.56
AAO diameter z-score	1.05	(0.94–1.22)	0.50
AAO CSA z-score	1.02	(0.94–1.29)	0.24
AAO stiffness measures			
Strain (%)	1.01	(0.99–1.02)	0.84
Distensibility (× 10^–3^ mmHg^−1^)	0.92	(0.84,1.02)	0.13
Distensibility z-score	0.97	(0.89–1.07)	0.55
β Stiffness Index	1.02	(0.98–1.06)	0.35
DAO stiffness measures			
Strain (%)	0.97	(0.94–0.99)	**0.03**
Distensibility (× 10^–3^ mmHg^−1^)	0.75	(0.63–0.89)	**0.001**
β Stiffness Index	1.07	(0.97–1.17)	0.19

Aortic stiffness parameters have been measured at 2 locations (AAO and DAO). Significant p values are in bold for logistic regression analysis

*AAO* Ascending aorta, *DAO* descending aorta, *CMR* Cardiac magnetic resonance, *CSA* cross-sectional area

**Table 4:** Multivariable analysis of factors associated with systemic hypertension

Parameter location	Odds ratio	95% CI	p Value
*Model 1*			
BAV	0.83	(0.42, 1.62)	0.59
Sex (male)	3.32	(1.66, 6.65)	**0.001**
Age at MRI (↑ 5 years)	1.32	(1.12, 1.54)	**0.001**
Age at CoA treatment (↑ 5 years)	1.11	(0.92, 1.34)	0.28
AAO distensibility *z*-score	1.08	(0.94, 1.24)	0.22
*Model 2*			
BAV	0.82	(0.39, 1.74)	0.61
Sex (male)	3.16	(1.48, 6.74)	**0.003**
Age at MRI (↑ 5 years)	1.25	(1.07, 1.46)	**0.004**
Age at CoA treatment (↑ 5 years)	1.02	(0.81, 1.28)	0.86
DAO Distensibility (× 10^–3^ mmHg^−1^)	1.01	(0.92, 1.1)	0.84

*DAO* descending aorta, *CMR* Cardiac magnetic resonance, *CSA* cross-sectional area

## Discussion

In this CMR study comparing aortic stiffness and hypertension prevalence in successfully repaired COA patients with or without a BAV, we found that although BAV patients have reduced AAO distensibility compared to those with a TAV, DAO distensibility and the prevalence of hypertension are similar in both groups.

### Aortic stiffness parameters

Prior studies have demonstrated increased AAO stiffness after successful COA repair [7, 25–28]. Similarly, patients with a BAV have been shown to have increased AAO stiffness [17, 18]. Because BAV is common in patients with COA, in this study we explored the hypothesis that the presence of BAV exacerbates AAO aortopathy in COA patients. Our results confirm this hypothesis by showing that patients with repaired COA who also have a BAV demonstrate reduced AAO distensibility compared to COA patients with a TAV. Several independent pathogenetic mechanisms for the increased aortic stiffness in COA and BAV have been proposed [26, 28–31]. It is likely that a coexistence of these pathogenetic mechanism in patients with both COA and BAV is responsible for the higher aortic stiffness seen in this group. In a smaller group of COA patients, Ghorbani et al. also recently reported that BAV was associated with lower AAO distensibility [32].

In contrast to AAO distensibility, DAO distensibility was not significantly different in COA patients with or without a BAV. This is consistent with prior reports showing that abnormalities in aortic stiffness are mostly confined to the AAO in both COA and BAV patients [17, 26]. In COA patients, this may be because the pre-stenotic AAO is subject to higher pressure prior to repair, while the DAO is protected from higher pressure. Similarly, BAV aortopathy and its other effects including dilation are mostly confined to the AAO, and therefore the DAO remains unaffected.

### Hypertension

Consistent with prior reports in successfully repaired COA patients, hypertension identified using current pediatric and adult practice guidelines was common (33%) in our study population. Despite the worse AAO distensibility in BAV patients, the prevalence of hypertension was similar in COA patients with or without a BAV. Further, in multivariable models exploring factors associated with hypertension, the presence of a BAV was not significantly associated with hypertension. These findings suggest that although the presence of BAV exacerbates the AAO aortopathy in COA patients, it does not contribute to hypertension. To our knowledge, this is the first study to examine this question. The only factors independently associated with hypertension were male sex and older age. It should however be noted that AAO distensibility in BAV patients deteriorates rapidly with aging, as previously reported by our laboratory [17]. Since our study cohort included mostly children and young adults, it is possible that in older patients with a stiffer AAO, BAV may play a more significant role in the pathogenesis of HTN. This requires further evaluation in future studies including older patients.

### Limitations

Several limitations of this work are worth considering. First, our study was limited by the retrospective study design. Second, although we used well established CMR methods for assessing aortic distensibility, it should be noted that peripheral systolic blood pressures may not accurately reflect central aortic pressure. Further, due to the retrospective study design, blood pressure was not measured simultaneous with aortic stiffness measurement.

## Conclusions

In young adults with adequately treated COA, those with BAV had a stiffer AAO compared to those with a TAV, but DAO stiffness was similar. In addition, prevalence of hypertension was not related to BAV. These results suggest that although the presence of a BAV in COA exacerbates the AAO aortopathy in a localized fashion, it does not exacerbate the more generalized vascular dysfunction and associated hypertension.

## Data Availability

The datasets during and/or analyzed during the current study are available from the corresponding author on reasonable request.
